# Hormonal Treatment of Transgender Women with Oral Estradiol

**DOI:** 10.1089/trgh.2017.0035

**Published:** 2018-05-01

**Authors:** Matthew C. Leinung, Paul J. Feustel, Jalaja Joseph

**Affiliations:** ^1^ Department of Medicine, Albany Medical College, Albany, New York.; ^2^ Department of Neuroscience and Experimental Therapeutics, Albany Medical College, Albany, New York.

**Keywords:** finasteride, oral estradiol, spironolactone, testosterone suppression, transgender woman

## Abstract

**Purpose:** Maintaining cross-sex hormone levels in the normal physiologic range for the desired gender is the cornerstone of transgender hormonal therapy, but there are limited data on how to achieve this. We investigated the effectiveness of oral estradiol therapy in achieving this goal.

**Methods:** We analyzed data on all transgender females seen in our clinic since 2008 treated with oral estradiol. We looked at the success of achieving serum levels of testosterone and 17-β estradiol in the normal range on various doses of estradiol (with and without antiandrogens spironolactone and finasteride).

**Results:** There was a positive correlation between estradiol dose and 17-β estradiol, but testosterone suppression was less well correlated. Over 70% achieved treatment goals (adequate 17-β estradiol levels and testosterone suppression) on 4 mg daily or more. Nearly a third of patients did not achieve adequate treatment goals on 6 or even 8 mg daily of estradiol. Spironolactone, but not finasteride, use was associated with impairment of obtaining desired 17-β estradiol levels. Spironolactone did not enhance testosterone suppression, and finasteride was associated with higher testosterone levels.

**Conclusions:** Oral estradiol was effective in achieving desired serum levels of 17-β estradiol, but there was wide individual variability in the amount required. Oral estradiol alone was not infrequently unable to achieve adequate testosterone suppression. Spironolactone did not aid testosterone suppression and seemed to impair achievement of goal serum 17-β estradiol levels. Testosterone levels were higher with finasteride use. We recommend that transgender women receiving estradiol therapy have hormone levels monitored so that therapy can be individualized.

## Introduction

Increasing numbers of individuals are seeking hormonal therapy for treatment of gender dysphoria.^1^ Clinical guidelines for therapy have been created, but these are not evidenced and rely instead upon clinical experience and are affected by regional regulations and reimbursement principles.^2–4^ The principle that cross-sex hormone levels be maintained in the normal physiologic range for the desired gender is the cornerstone of therapy, but there are not a lot of data on how best to achieve this.^3,4^

We created a database in 2003 of patients seen in our transgender clinic and have collected demographic and treatment information since that time.^1^ We began using oral 17-β estradiol instead of ethinyl estradiol and conjugated estrogens around 2006–2007 due to safety concerns over deep vein thrombosis. This enabled us to begin collecting data on 17-β estradiol levels. For this report, we analyzed our database to determine the effect of our therapy with oral estradiol and antiandrogens on serum 17-β estradiol and testosterone levels.

## Methods

Our clinic is an academic referral center in upstate New York. We analyzed data on all transgender females seen in our clinic since 2007 treated with oral estradiol. At each visit, data for estrogen (form and dose) and antiandrogen type and dose were recorded. Serum levels of 17-β estradiol and total testosterone, when assayed, were recorded as well (lack of insurance coverage limited the frequency of these determinations for many patients). This activity was approved by the Institutional Review Board of Albany Medical Center Hospital. Total testosterone was determined by chemiluminescent immunoassay (Access Testosterone assay, Beckman Coulter, Inc., CA). In 2013, the Albany Medical Center Clinical Chemistry department, in conjunction with the Division of Endocrinology, set internal reference ranges for this assay as follows: normal male >320 ng/dL; equivocal 200–319 ng/dL; and hypogonadal <200 ng/dL. The female normal reference range was set at 10–75 ng/dL.^5^ The 17-β estradiol levels were determined by chemiluminescent microparticle immunoassay (Architect Estradiol assay, Abbott Laboratories, IL). Normal male reference range is 11–44 pg/mL, and normal menstruating female follicular phase reference range is 21–251 pg/mL. The Clinical Chemistry Laboratory at Albany Medical Center is certified for estradiol testing by New York State Department of Health who accepts College of American Pathology proficiency as an index of performance.

Our approach for the initiation of hormonal therapy in transgender women is to start at lower doses of estradiol (e.g., 2 mg oral daily) and titrate upwards over the course of a year.^1^ This is done based on the experience of the induction of puberty in natal girls in whom rapid estrogen exposure was found to lead to premature breast bud fusion and poor breast development.^6^ Serum hormone levels are measured no sooner than 3 months after dose initiation or change. Antiandrogen therapy (as finasteride 5 mg daily or spironolactone 100 mg twice daily) is offered at the initiation of therapy to help decrease secondary sexual hair growth, without regard to serum testosterone levels. The estradiol dose is titrated upwards with the goal of obtaining estradiol levels above 100 but below 200.^3^ If testosterone levels are not adequately suppressed (to below 100) while 17-β estradiol levels are at goal, we typically add medroxyprogesterone acetate daily (2.5–10 mg orally). Individual circumstances (age, patient preference, and comorbidities) are taken into consideration leading to some variation in this approach (e.g., use of transdermal estrogen, slower dose titration, or not proceeding to higher doses).

Serum 17-β estradiol and testosterone (T) levels were evaluated by multiple regression as functions of estrogen dose, spironolactone (absent or present), and finasteride (absent or present). All two-way interactions were also tested and included in the final model if significant. Data included all available data points in all patients (analyses were also done using only the first or the last data point in each patient with essentially similar results although some effects failed to reach statistical significance, which may have been due to reduced numbers of points).

In addition, the odds of success of the therapy defined as 17-β estradiol >100 pg/mL were assessed by multiple logistic regression. A separate analysis for T<100 ng/dL was also performed. Independent variables were estrogen dose (in mg/day), spironolactone (absent or present), finasteride (absent or present), and all two-way interactions with results reported for models restricted to significant predictors.

Serum 17-β estradiol level and body mass index (BMI) were evaluated by linear regression. The effect of BMI on estradiol dosing was assessed by analysis of variance at three doses (4, 6, and 8 mg daily).

All data were analyzed using Minitab statistical software with significance accepted at *p*<0.05.

## Results

From 2007 through 2016, 184 transgender women were seen in our clinic and received hormonal therapy. Of these, 13 were treated with nonoral estrogen, 4 did not have 17-β estradiol levels determined, and one adolescent patient had not progressed beyond GnRH suppression. This left 166 women with an average age at our initial visit of 36.8±13.4 years (range 10.5–74.1). The average BMI was 28.2±6.8 (range 16.7–57.6), with 43 (25.9%) having a BMI greater than 30 (Table 1). In 10 women we only had postoperative values. This left 156 individuals (adults over 18 years of age) with corresponding testosterone and 17-β estradiol values while on oral estradiol, many of whom had multiple determinations (including at different doses). Twenty individuals did not progress beyond 2 mg/day dose of estradiol. This left 136 patients who progressed to 4 mg oral estradiol daily: 21 (15.4%) of these achieved treatment goals (17-β estradiol level >100 and or suppression of testosterone). Thirty eight (27.9%) did not achieve treatment goals even on 6 or 8 mg and received progesterone. In 11 (29%) patients serum testosterone levels still did not drop below 100 even with the addition of medroxyprogesterone acetate in doses of 2.5–10 mg. A total of 25 (18.3%) individuals did not achieve adequate 17-β estradiol levels on 6 mg estradiol daily and were increased to 8 mg daily (with 13 of those, 9.6%, still not reaching goal).

**Table 1.** **Patient Demographics**

	Number	Range
No. of male to female transgender	166	
Age at initiation of treatment in years	36.8±13.4	13.1–74.9
BMI	28.2±6.8	16.7–57.6
BMI >30	43 (25.9%)	
HIV Positive	6 (3.4%)	
No. of patients on finasteride	49 (29.5%)	
No. of patients on spironolactone	61 (36.7%)	
Patients who had started treatment before initial visit	59 (35.5%)	
Duration of Therapy^a^ in years	7.5±7.1	0.3–34.5
Duration of Estradiol Therapy^b^ in years	4.3±3.1	0.3–10.5
No. receiving ≥4 mg estradiol daily, with laboratories	136	
No. achieving goal at 4 mg daily	21 (15.4%)	
No. achieving goal	77 (56.7%)	
No. of patients needing medroxyprogesterone	38 (27.9%)	2.5–10 mg

^a^ Mean duration of hormonal therapy until vaginoplasty/orchiectomy or last laboratory determination, in years (includes therapy before 2007).

^b^ Mean duration of therapy on oral estradiol until last laboratory determination, in years.

BMI, body mass index.

**FIG. 1.**
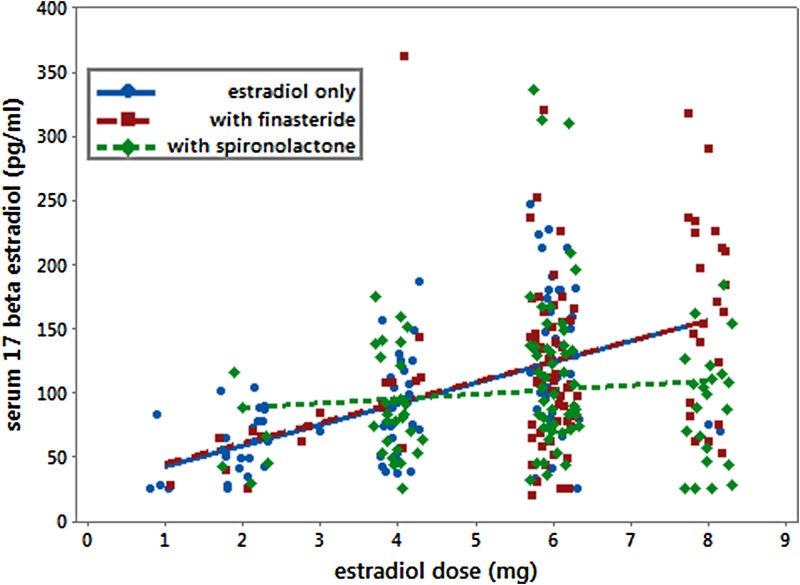
Serum 17-β estradiol versus estradiol dose. Serum 17-β estradiol levels while on estradiol only are represented by circles (blue): values obtained while on finasteride as well are represented by squares (red), and values obtained while on spironolactone are represented by diamonds (green).

17-β estradiol was increased by 16.3 (SE=2.1; *p*<0.001) pg/mL for each mg increase in estradiol dose (Fig. 1). Spironolactone had both a main effect and also interacted with estradiol reducing the slope. The presence of spironolactone reduced the effectiveness of estradiol doses reaching desired serum 17-β levels by reducing the slope by 11.6 (SE=3.7; *p*=0.002) pg/mL for each mg increase in estradiol in addition to having a main effect of increasing 17-β estradiol by 45.9 (SE=20.9; *p*=0.03) pg/mL. Adjusted model r-squared was 0.17.

Success in achieving a 17-β estradiol >100 pg/mL was evaluated by logistic regression. Interactions were not significant. Odds of success increased by a factor of 1.50 (95% confidence interval [CI] 1.30–1.74) for each 1 mg increase in estradiol dose. The presence of spironolactone reduced the odds of success by a factor of 0.52 (95% CI: 0.29–0.91). Finasteride had no significant effect (odds ratio: 0.90; 95% CI: 0.51–1.60).

Testosterone was decreased by 8.7 (SE=3.8; *p*=0.21) ng/dL for each mg increase in estradiol dose. There were no significant interaction effects, and spironolactone had no statistically significant effect on testosterone (testosterone increased 10.6 ng/dL (SE=16; *p*=0.5). The presence of finasteride elevated serum testosterone by 91.7 (SE=15.8; *p*<0.001) ng/dL. Adjusted model r-squared was 0.11.

Success in achieving a serum testosterone level <100 ng/dL was evaluated by logistic regression. Interactions were not significant. Odds of success increased by a factor of 1.23 (95% CI: 1.08–1.40) for each 1 mg increase in estradiol dose. The presence of finasteride reduced the odds of success by a factor of 0.27 (95% CI: 0.15–0.46). Spironolactone had no significant effect (odds ratio: 0.75; 95% CI: 0.44–1.29).

**FIG. 2.**
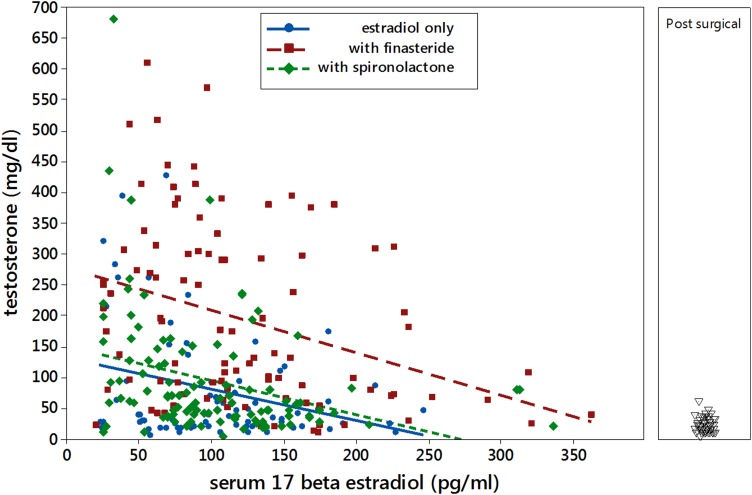
Serum testosterone versus serum 17-β estradiol level. Serum testosterone levels while on estradiol only are represented by circles (blue): values obtained while on finasteride as well are represented by squares (red), and values obtained while on spironolactone are represented by diamonds (green). The panel on the right shows values for individuals obtained after surgery (vaginoplasty and/or orchiectomy).

Regression of testosterone as a function of measured serum 17-β estradiol (Fig. 2) found significant effects of 17-β estradiol (slope=−0.462 mg/dL testosterone ng/dL 17-β estradiol; *p*=0.012). The presence of finasteride raised testosterone by 126 ng/dL (SE=25; *p*<0.001), and spironolactone had no significant effect (*p*=0.51). Adjusted r-squared=0.21.

We also looked at serum levels of testosterone and 17-β estradiol in postoperative transgender women (Fig. 2, panel on right). There were 44 serum testosterone determinations in 26 different individuals. Mean testosterone level was 21.7 ng/dL with a standard deviation of 12.4 ng/dL (13 determinations were below 10 ng/dL, the lower limit of detection for the assay). Presence or absence of surgery had no effect on the level of serum 17-β estradiol on different doses of estradiol.

**FIG. 3.**
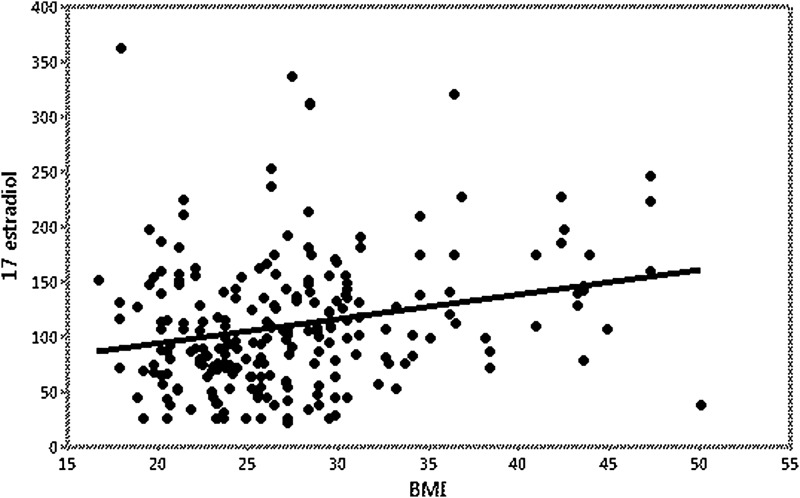
Serum 17-β estradiol versus BMI. There was a positive association of BMI with serum 17-β estradiol level, but the variance attributable to BMI was small (r-squared=0.056). BMI, body mass index.

We examined the effect of obesity on 17-β estradiol levels. There was no statistically significant difference in BMI between the three main dosages (4, 6, and 8 mg daily). BMI did have an influence on 17-β estradiol levels (*p*<0.001, levels increased by 2.2 (SE=0.6) units for each unit increase in BMI), but the variance attributable to BMI was small (r-squared=0.056) (Fig. 3). There was no evidence that the dose affected the relationship between BMI and 17-β estradiol levels (*p*=0.35; interaction term in multiple regression with the effects of dose and BMI).

Age had no effect on the ability to achieve suppression of testosterone at various doses or the need for medroxyprogesterone acetate.

## Discussion

The goal of hormonal therapy for transgender individuals is to induce the physical changes commensurate with the desired gender. The cornerstone of therapy, as expressed by the Endocrine Society, is to maintain sex steroid levels in the normal physiologic range for that gender. Clinical experience has provided some guidance as to how to achieve this, but data are limited. We reviewed our experience over the past 10 years of using oral estradiol with and without antiandrogens (spironolactone and finasteride) and progestin (medroxyprogesterone acetate).

We found a lot of variability in the response of individuals to various doses of estradiol. There was a clear not unexpected positive correlation of increasing doses and serum levels of 17-β estradiol. However, while some achieved goal levels of serum 17-β estradiol (15.4%) on 4 mg daily dose, 18.3% needed to progress to 8 mg daily (with half of those still not achieving goal). We also found an expected negative correlation between serum level of 17-β estradiol and serum testosterone. However, there was again a lot of variability, and 28% of individuals needed the addition of medroxyprogesterone to get further testosterone suppression. Since aromatization of androgens to estrogens occurs in adipose tissue, we examined the effect of BMI on 17-β estradiol. There was a positive association with BMI and 17-β estradiol levels, but the impact was small. BMI did not have any effect on estradiol dosing.

The reason for this variability in response is unclear. Concomitant medication use could be a factor affecting the metabolism of sex steroids, but we believe this could have affected at most only a small percentage of our sample. Adherence to therapy could also be a factor, but we find this population to be generally very well motivated, and we routinely ask about missing doses or taking additional (not prescribed) agents.

Recommendations for hormonal therapy include antiandrogen therapy. In the United States, cyproterone acetate is not available, and GnRH treatment is very expensive and not covered by insurance. Spironolactone is often used since at high doses it blocks the androgen receptor. It has also been reported to decrease testosterone production and also is reported to have some agonist effect at the estrogen receptor.^7^ However, there are very little data demonstrating the testosterone lowering effect of spironolactone in transgender women. Prior et al. in 1989 reported that in 27 transgender women receiving “high dose” conjugated estrogen who were switched to spironolactone (dosed at 200–600 mg daily, more than the 200 mg/day recommended by the Endocrine Society treatment guidelines) and a cyclic hormone regimen, mean testosterone levels fell from 161 to 87 ng/dL.^8^ Included in this regimen was medroxyprogesterone acetate, 10 mg daily for at least 2 weeks out of 4 (some received continuous medroxyprogesterone “to aid in testosterone reduction”). This is the article cited in support of spironolactone suppressing testosterone, when it supports just as well the use of medroxyprogesterone. In studies examining spironolactone use in the treatment of hirsutism, no change in baseline testosterone levels has been found (although cyproterone acetate, a progestational compound, did lead to a decrease).^9,10^ We found no difference in testosterone levels at equivalent levels of estradiol when spironolactone was part of the regimen, implying no additional effect of spironolactone on testosterone production. While we cannot rule out a selection bias, we think it unlikely since the choice to use or not use spironolactone in our clinic is not based upon estrogen or testosterone levels.

There are little data to guide the selection of antiandrogen therapy. We offer finasteride or spironolactone to patients primarily to help decrease face and body hair. In older patients or those with medical comorbidities we tend to recommend finasteride over spironolactone due to potential electrolyte effects. Concern has been raised about the potential of finasteride to cause depression and sexual dysfunction through its decrease in 5 dihydrotestosterone. However, these data pertain to cisgender men who presumably do not desire losing the actions of testosterone, not to transgender women.^11^ Conversely, spironolactone may have effects on depression as well.^12^ The desired outcomes of hormonal therapy are the physical manifestations associated with feminine escutcheon (breast development, female fat distribution, decreased facial and body hair, and skin softening). Finasteride performs as well as spironolactone in clinical trials for treatment of hirsutism.^10,13^ We are aware of one report addressing breast development and antiandrogen use. Seal et al., reported that among transgender women seeking breast augmentation (implying dissatisfaction with the results of hormonal therapy), the type of estrogen used did not seem to matter.^14^ However, spironolactone use (but not finasteride or cyproterone) was more common in those seeking augmentation. Our somewhat surprising finding of lower estrogen levels at recommended treatment doses of estradiol while on spironolactone could be a factor. We have no explanation for this finding. Mention has been made of possible agonistic effect of spironolactone at the estrogen receptor.^3,4^ The report cited for this actually found that in the absence of estrogen, spironolactone acted as an agonist, but in the presence of estrogen it behaved as a competitive inhibitor.^7^ If this is in fact the case, it could have a negative impact on breast development.

Finasteride is known to increase circulating testosterone levels in men by 8–10%.^15^ Our transgender women receiving finasteride did exhibit higher levels of testosterone than those who did not. The increase we saw was much higher than 10%. This no doubt increased the number of individuals not achieving the testosterone suppression goal. Since finasteride blocks testosterone effects in testosterone sensitive tissue, it is uncertain if this would have a deleterious effect. We were unable to assess whether finasteride use had any impact on hormone induced physical changes.

We had chosen a suppressed testosterone level as below 100. This is higher than the Endocrine Society recommendations and higher than the normal female range in our assay.^3^ The level was chosen early based upon our experience with the performance of the assay and consideration of the ability of estrogen to raise sex hormone binding globulin, thus elevating total testosterone levels. Such an effect was recently reported by Deutsch et al. where total testosterone levels of 10 of 15 transgender women on estradiol did not suppress into the normal female range, but in 14 of 15 the free testosterone did.^16^ The choice of estrogen levels between 100 and 200 was based upon the Endocrine Society guidelines and the normal male range in our assay.^3^

Even with a liberal goal, a third of our patients did not achieve the desired testosterone suppression despite reaching adequate serum 17-β estradiol levels. Some of this occurred in patients receiving finasteride, where we found a higher than expected response of increased testosterone levels. As stated above, the significance of this is uncertain. There are limited data but a general thought is that estrogen alone is often insufficient to fully suppress serum testosterone levels.^4^ Before the early 2000's, conjugated estrogens, not estradiol, were the principal form of administered estrogen. In the report by Deutsch et al. of 16 transgender women receiving estrogen therapy (14 through sublingual route and 2 through intramuscular injection) for 6 months, total testosterone was suppressed in only 10, although free testosterone was suppressed in 15.^16^ It should be noted that a quarter of their patients had baseline testosterone levels below normal, and the mean 17-β estradiol level was 258 pg/mL (above the level recommended by the Endocrine Society guidelines) with 3 attaining a level in the “supraphysiologic” range of greater than 498 pg/mL.^16^

Since the initial submission of this article a study has been published on testosterone suppression in transgender women in another US cohort. Liang et al. reviewed testosterone and 17-β estradiol levels in 87 transgender women treated with oral estrogens (67 on oral estradiol) and spironolactone.^17^ They report no correlation between estradiol dose and estradiol level and no correlation between estradiol level and testosterone level. This is contrary to our findings, where there was a correlation. One would expect there to be an inverse correlation between estradiol levels and testicular function, given our understanding of the normal function of the hypothalamic-pituitary-gonadal axis. This difference could be due to sample size, as we saw wide variability in the response or to methodology (they broke down patients into quartiles for analysis). As with our findings, they found that over a quarter of their subjects were unable to achieve adequate testosterone suppression with estrogen (and spironolactone).^17^ Our numbers may have been slightly higher due to the use of finasteride and its effects on measured testosterone level (see Results, Figure 2).

The report by Liang is interesting in another respect. They gave all patients spironolactone and thus could not report on estradiol use alone. However, they found no association of spironolactone dose and testosterone suppression.^17^ As stated above, the data supporting direct suppression of testosterone production by spironolactone are tenuous at best. A lack of a dose ranging effect on testosterone levels lends no further support to this.

It may be advisable to reexamine the role of spironolactone in the hormonal care of transgender women. While it clearly has shown benefit regarding hirsutism, our finding of the lack of an independent effect on testosterone levels (and no supporting data from the study of Liang et al), our additional finding of lower estrogen levels at recommended treatment doses of estradiol, the report of a greater rate of breast augmentation in spironolactone users compared to nonusers,^14^ and the sparse and contradictory mechanistic reports,^7,8^ all point to the need for further study.

One limitation of this study is the testosterone assay. Tandem mass spectrometry is considered the best method for assessing serum testosterone, but this assay is more expensive.^5^ Medical care for transgender individuals has only recently been mandated to be covered by insurance in New York State, so we had many patients that found even the less expensive assay difficult to afford. Another reason to not change the assay is that we feel confident in our assay's performance. The most problematic area for testosterone assays is in discriminating low normal from mild to moderately low levels (i.e., 200–300 pg/mL).^5^ We reviewed the performance of our assay in 2013 and revised our reference ranges. This assay is clearly able to distinguish very low (postorchiectomy) levels from suppressed levels and normal levels from suppressed levels.

Another limitation of the study was that it was not prospective. While the therapeutic approach has been standard over the past 10 years, there is ample room for individualization of therapy. We believe the relatively large sample size overcomes this issue.

In summary, we found that oral estradiol was effective in achieving the desired serum levels of 17-β estradiol in 90% of patients, but there is wide individual variability in the dose required. Testosterone suppression is at times difficult to achieve even with doses of estradiol that reach desired levels of serum 17-β estradiol. Since GnRH analogs and cyproterone are not readily available to us, we added medroxyprogesterone and found some success obtaining further testosterone suppression. We unexpectedly did not find that spironolactone aided in testosterone suppression and further found that it seemed to impair the ability to reach desired serum 17-β estradiol levels. While finasteride did not impact 17-β estradiol levels, total testosterone levels were generally higher, although the impact on physical outcomes is uncertain. We recommend that transgender women receiving estradiol therapy have hormone levels monitored so that therapy can be individualized.

## Abbreviations Used

BMI: body mass index
CI: confidence interval
